# Early evaluation of tumour metabolic response using [^18^F]fluorodeoxyglucose and positron emission tomography:a pilot study following the phase II chemotherapy schedule for temozolomide in recurrent high-grade gliomas

**DOI:** 10.1054/bjoc.1999.0971

**Published:** 2000-01-18

**Authors:** C S Brock, H Young, S M O'Reilly, J Matthews, S Osman, H Evans, E S Newlands, P M Price

**Affiliations:** CRC-PET Oncology Group, MRC Cyclotron Unit, Hammersmith Campus, Imperial College of Science and Medicine, Du Cane Road, London, W12 0HS, UK; Department of Medical Oncology, Charing Cross Hospital, Fulham Palace Road, London, W6, UK

**Keywords:** temozolomide, positron emission tomography, glioma, metabolic rate of glucose (MRGlu)

## Abstract

Quantitation of metabolic changes in tumours may provide an objective measure of clinical and subclinical response to anticancer therapy. This pilot study assesses the value of quantitation of metabolic rate of glucose (MRGlu) measured in mmol min^−1^ml^−1^to assess early subclinical response to therapy in a relatively non-responsive tumour. Nine patients receiving the CRC Phase II study schedule of temozolomide were assessed with [^18^F]fluorodeoxyglucose ([^18^F]FDG) dynamic positron emission tomography (PET) scans prior to and 14 days after treatment with temozolomide given as 750–1000 mg m^−2^over 5 days every 28 days. Tumour MRGlu was calculated and compared with objective response at 8 weeks. Pretreatment MRGlu was higher in responders than non-responders. The responding patient group had a greater than 25% reduction in MRGlu in regions of high focal tumour uptake (HFU). Whole tumour changes in MRGlu did not correlate with response. Percentage change in HFU standardized uptake value (SUV) did discriminate the responding from the non-responding patients, but not as well as with MRGlu. Large differences also occurred in the normal brain SUV following treatment. Thus, MRGlu appeared to be a more sensitive discriminator of response than the simplified static SUV analysis. Changes in MRGlu may reflect the degree of cell kill following chemotherapy and so may provide an objective, quantitative subclinical measure of response to therapy. © 2000 Cancer Research Campaign


British Journal of Cancer (2000) **82**(3), 608–615
© 2000 Cancer Research Campaign
DOI: 10.1054/ bjoc.1999.0971, available online at http://www.idealibrary.com on IDEAL®

### Early evaluation of tumour metabolic response using [$^{18}$F]fluorodeoxyglucose and positron emission tomography: a pilot study following the phase II chemotherapy schedule for temozolomide in recurrent high-grade gliomas

**CS Brock[1], H Young[1], SM O'Reilly[1,*], J Matthews[1], S Osman[1], H Evans[2], ES Newlands[2] and PM Price[1]**

[1]CRC-PET Oncology Group, MRC Cyclotron Unit, Hammersmith Campus, Imperial College of Science and Medicine, Du Cane Road, London W12 0HS, UK; [2]Department of Medical Oncology, Charing Cross Hospital, Fulham Palace Road, London W6, UK

**Summary** Quantitation of metabolic changes in tumours may provide an objective measure of clinical and subclinical response to anticancer therapy. This pilot study assesses the value of quantitation of metabolic rate of glucose (MRGlu) measured in mmol min$^{-1}$ ml$^{-1}$ to assess early subclinical response to therapy in a relatively non-responsive tumour. Nine patients receiving the CRC Phase II study schedule of temozolomide were assessed with [$^{18}$F]fluorodeoxyglucose ([$^{18}$F]FDG) dynamic positron emission tomography (PET) scans prior to and 14 days after treatment with temozolomide given as 750–1000 mg m$^{-2}$ over 5 days every 28 days. Tumour MRGlu was calculated and compared with objective response at 8 weeks. Pretreatment MRGlu was higher in responders than non-responders. The responding patient group had a greater than 25% reduction in MRGlu in regions of high focal tumour uptake (HFU). Whole tumour changes in MRGlu did not correlate with response. Percentage change in HFU standardized uptake value (SUV) did discriminate the responding from the non-responding patients, but not as well as with MRGlu. Large differences also occurred in the normal brain SUV following treatment. Thus, MRGlu appeared to be a more sensitive discriminator of response than the simplified static SUV analysis. Changes in MRGlu may reflect the degree of cell kill following chemotherapy and so may provide an objective, quantitative subclinical measure of response to therapy. © 2000 Cancer Research Campaign

**Keywords:** temozolomide; positron emission tomography; glioma; metabolic rate of glucose (MRGlu)

The ability to assess tumour response after a single cycle of chemotherapy may improve patient management and early evaluation of new anticancer therapies. Quantitative tumour metabolic response rate assessment has been suggested as a surrogate end point for tumour response to therapy and may assist in the identification and scheduling of new chemotherapeutic strategies in phase I and II clinical trials (Price and Jones, 1995). Clinically, early identification of those patients with non-responding tumours may permit the cessation of ineffective therapy and promote individualization of therapy.

Positron emission tomography (PET) is an in vivo functional imaging modality using positron-emitting radiolabelled compounds. The most widely used radiotracer in oncology is 2-[$^{18}$F]fluoro-2-deoxy-D-glucose ([$^{18}$F]FDG), a glucose analogue. It is transported by facilitated diffusion into the cells where it is phosphorylated by hexokinase to form [$^{18}$F]fluorodeoxyglucose-6-phosphate ([$^{18}$F]FDG-6-P). It effectively becomes 'trapped' intracellularly since dephosphorylation is slow especially in tissues with low levels of glucose-6-phosphatase. [$^{18}$F]FDG uptake is enhanced in tumours due to both increased transport and phosphorylation (Bennett et al, 1978; Herholz et al, 1992; Brown et al, 1993, 1996). Increased [$^{18}$F]FDG uptake, as measured by PET, although a function of proliferative activity (Minn et al, 1988*a*; Okada et al, 1992; Higashi et al, 1993) is related to the number of viable tumour cells (Herholz et al, 1993). Therefore, reduction in the viable tumour cell population with effective chemotherapy should result in reduced [$^{18}$F]FDG uptake.

A number of pilot studies have shown that early reduction in tumour [$^{18}$F]FDG uptake is related to tumour response to effective chemotherapy in extracerebral tumours (Minn et al, 1988*b*; Ichiya et al, 1991; Wahl et al, 1993; Jansson et al, 1995). Pilot studies with intracerebral tumours have shown reduced tumour [$^{18}$F]FDG uptake corresponding with radiological improvement and therapeutic response duration in patients with medulloblastoma (Holthoff et al, 1993). For patients with gliomas, reduction in tumour [$^{18}$F]FDG uptake corresponded with radiological response 30 days after combination chemotherapy (Rozental et al, 1989) and with patient survival (De Witte et al, 1994).

Assessment of alterations in tumour extent with therapy using computerized tomography (CT) and magnetic resonance imaging (MRI) can be a problem for gliomas where the tumour can not be clearly delineated from post-operative enhancement (Cairncross et al, 1985) and radiation-induced necrosis (Patronas et al, 1982). Interpretation of sequential anatomical scans to monitor treatment response in brain tumours can be complicated. The addition of a metabolic response marker in the form of [$^{18}$F]FDG-PET may improve response assessment in these tumours.

*Received 3 February 1998*
*Revised 11 June 1999*
*Accepted 8 July 1999*

*Correspondence to: PM Price*

Present address: Clatterbridge Hospital, Clatterbridge Road, Wirral, Merseyside L63 4JY, UK

Increased [$^{18}$F]FDG has been shown in gliomas and correlated with tumour grade (Di Chiro et al, 1982) and prognosis (Alavi et al, 1988). The [$^{18}$F]FDG-PET technique has been used to differentiate tumour recurrence from necrosis (Valk et al, 1988). It has been proposed that changes in [$^{18}$F]FDG uptake could be used to parallel phase I and II clinical trials (Price and Jones, 1995) to provide an objective quantitative measure of clinical and subclinical response.

Temozolomide is a methylating imidazotetrazinone which was evaluated during a phase II clinical trial at Charing Cross Hospital, London, under the auspices of the Cancer Research Campaign's (CRC) Phase I & II Clinical Trials Committee. The initial phase I trial had previously demonstrated that the drug had activity against gliomas, malignant melanoma and mycosis fungoides on a five day schedule repeated every 28 days, with myelosuppression being the main toxicity (Newlands et al, 1992; O'Reilly et al, 1993).

The aim of this pilot study was to evaluate [$^{18}$F]FDG-PET as an early tumour metabolic response marker in high-grade gliomas treated with the phase II treatment schedule of temozolomide.

### MATERIALS AND METHODS

### Patients

Nine patients with recurrent grade III and IV gliomas being treated using the CRC Phase II trial schedule of temozolomide were recruited for the [$^{18}$F]FDG-PET study. Eligibility criteria included those for entry into the phase II clinical study (O'Reilly et al, 1993) and the ability to undergo two PET scans. No patient had received radiotherapy or chemotherapy in the 4 weeks preceding treatment with temozolomide, or in the preceding 6 weeks for those who had received treatment with nitrosoureas. All had evaluable tumour on CT scan and had WHO performance status of ≤3.

Temozolomide was administered at a dose of 750 mg m$^{-2}$ divided equally over 5 days for the first cycle, increased to 1000 mg m$^{-2}$ divided over 5 days for the subsequent cycles providing there had been no myelosuppression noted on day 22. The cycles were repeated every 28 days (O'Reilly et al, 1993).

All the patients gave informed written consent for this study as approved by the Hammersmith Hospitals Research and Ethics Committee and UK Administration of Radioactive Substances Advisory Committee.

### [$^{18}$F]FDG-PET imaging

[$^{18}$F]FDG-PET scans were performed at the Medical Research Council (MRC) Cyclotron Unit, prior to the first treatment cycle with temozolomide, and a repeat scan performed within 14 days of completing the first 5-day oral course. One patient (no. 1) had both [$^{18}$F]FDG-PET scans performed 14 days apart prior to commencing temozolomide therapy, to act as a control subject in order to determine reproducibility of data collection and analysis.

Patients were fasted for 4–6 h prior to the PET scan, in order to stabilize their blood glucose concentration and enhance tumour [$^{18}$F]FDG uptake (Lindholm et al, 1993; Ishizu et al, 1994). For those receiving corticosteroids, dexamethasone dosage was stabilized for a minimum of 2 weeks prior to performing the baseline scans and initiating temozolomide to minimize changes in oedema induced by steroid administration (Cairncross et al, 1988). There was no alteration in the patients' dose of corticosteroids nor anticonvulsant medication until at least after the second [$^{18}$F]FDG-PET study. This is important as dexamethasone can elevate circulating plasma glucose levels which may confound the interpretation of SUV and $K_1$ (Roelcke et al, 1998) and it has also been shown to decrease normal cerebral glucose metabolism (Fulham et al, 1995).

[$^{18}$F]FDG was synthesized on-site at the MRC Cyclotron Unit, using the stereospecific reaction consisting of a nucleophilic fluorination followed by a de-protection stage to produce a no-carrier added FDG (Hamacher et al, 1986). The radiochemical purity of the [$^{18}$F]FDG was 100%. The studies were performed at a time when [$^{18}$F]FDG was assessed only for Kryptofix$^{R}$, a toxic catalyst and impurity.

A peripheral venous cannula was inserted for intravenous administration of [$^{18}$F]FDG. A second cannula was inserted into the patient's contralateral radial artery, having performed Allen's test, to measure arterial [$^{18}$F]FDG and glucose concentrations.

All image data were acquired on an ECAT 953B CTI Neuro-PET scanner (CTI/Siemens, Knoxville, TN, USA). The patients were positioned using a head support and the orbito-meatal line parallel to the transaxial plane of the tomograph, such that the tumour position was well within the 10.8 cm field of view. Prior to the [$^{18}$F]FDG injection, a transmission scan with $^{68}$Ge rod sources was performed to measure and correct for tissue attenuation of 511 keV photons. Emission scanning was commenced 30 s prior to [$^{18}$F]FDG administration with a protocol of 23 frames (6 × 30 s; 7 × 60 s; 10 × 5 min). Data acquisition was in the two-dimensional mode.

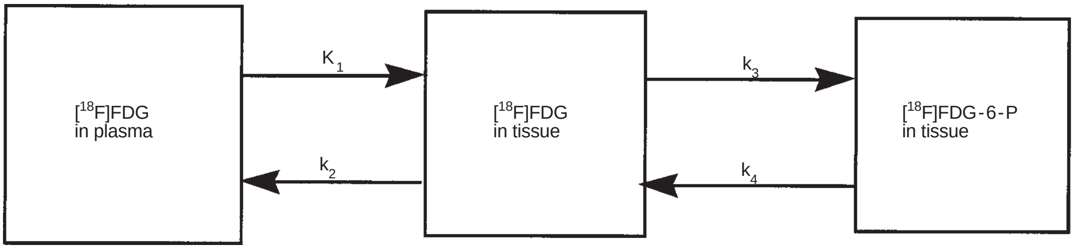

**Figure 1**. Diagram demonstrating the metabolism of [$^{18}$F]FDG in tissue. The rate constants $K_1$, $k_2$, $k_3$ and $k_4$ are determined using kinetic modelling and characterize delivery, washout, phosphorylation and dephosphorylation respectively. The original three compartment model (Sokoloff et al, 1977) has been extended to include a dephosphorylation rate constant ($k_4$) of the reaction [$^{18}$F]FDG-6-P to [$^{18}$F]FDG (Phelps et al, 1979; Huang et al, 1980). The rate constants ($K_1$, $k_2$, $k_3$, $k_4$ min$^{-1}$), in Figure 1 reflecting transfer between compartments, are estimated from the model, using a non-linear fitting algorithm, the measured tissue time activity curves and the input function.

© 2000 Cancer Research Campaign 

**Table 1** MRC neurological status

| | |
|---|---|
| 0= | No neurological deficit. |
| 1= | Some neurological deficit but function adequate for useful work. |
| 2= | Neurological deficit causing moderate functional impairment, e.g. able to move limb(s) only with difficulty, moderate dysphasia, moderate paresis, some visual disturbances (e.g. field defect). |
| 3= | Neurological deficit causing major functional impairment, e.g. inability to move limbs, gross speech or visual disturbance. |
| 4= | No useful function – inability to make conscious responses. |

**Table 2** Patient clinico-pathological details

| Patient | Sex | Age (years) | Initial tumour grade | Steroid therapy | Anti-convulsant therapy |
|---|---|---|---|---|---|
| 1 | Female | 44 | IV | Yes | Yes |
| 2 | Male | 48 | IV | No | Yes |
| 3 | Male | 28 | III | Yes | No |
| 4 | Female | 55 | IV | Yes | No |
| 5 | Male | 43 | III | Yes | Yes |
| 6 | Male | 33 | II | Yes | Yes |
| 7 | Female | 52 | II | No | Yes |
| 8 | Female | 48 | IV | Yes | Yes |
| 9 | Male | 34 | III | Yes | No |

The [$^{18}$F]FDG was injected as an intravenous bolus given over a 30 s period starting 30 s into the [$^{18}$F]FDG-PET study. The patient's radial arterial blood was continuously withdrawn over a calibrated bismuth germinate (BGO) detector allowing continuous measurement of arterial radioactivity (Ranicar et al, 1991). Downstream from the detector discrete samples were taken immediately prior to the start of the scan and at 5, 10, 20 and 50 min. These samples were used to measure circulating glucose levels and partition [$^{18}$F] radioactivity between whole blood and plasma.

### PET data analysis

The PET data were attenuation corrected and reconstructed using filtered back projection with a Hann filter, cut off at the Nyquist frequency (8.2 ± 0.2 mm), into two-dimensional matrices of 128 × 128 with pixel dimensions of 2.016 × 2.016 mm. The 31 slices were stacked to form a three-dimensional volume with an axial slice width of 3.375 mm.

The second [$^{18}$F]FDG-PET study was co-registered to the first using the Wood's algorithm (Woods et al, 1992). All frames for each study were summed to provide an image of higher statistical quality for region of interest (ROI) drawing. ROI analysis was performed using the Sunview package 'Analyze' (Analyze, Mayo Clinic, Rochester, MN, USA).

### Region of interest definition

Gliomas are heterogeneous structures containing oedema, scar tissue, necrosis as well as viable tumour cells. Clear delineation of the tumour margins from normal brain tissue and peripheral oedema can prove difficult using anatomical imaging. In this study, ROIs were defined with visual reference to a pretreatment CT scan to assist in the anatomical localization of tumour extent. Normal brain (B) was defined as uninvolved contralateral temporo-parietal white matter, whole tumour (WT) defined a region encompassing the whole tumour and high focal [$^{18}$F]FDG uptake areas (HFU) were defined as a small area within the tumour with the highest uptake defined visually. ROIs for all three tissue areas are defined on the baseline [$^{18}$F]FDG-PET study. Co-registration of second to the first [$^{18}$F]FDG PET study was employed (Woods et al, 1992). Tissue time activity curves were generated by applying the ROIs defined to the co-registered dynamic PET scan frames for both studies. The average count per pixel was used for each ROI. Where it was possible to define more than one HFU, the one with the highest MRGlu at baseline was used for comparison.

### Calculation of MRGlu

The [$^{18}$F]FDG plasma time concentration curve was generated from the continuous and discrete arterial blood samples to provide an input function. The metabolic rate for glucose (MRGlu μmol min$^{-1}$ ml$^{-1}$) was determined using the arterial input function, and the PET-generated tissue time activity curves with a three compartment model (Figure 1).

The tissue or tumour glucose utilization rate is given by:

$$\text{MRGlu } (\mu\text{mol min}^{-1}\text{ ml}^{-1}) = \frac{C_{pl}}{LC} \times \frac{K_1 k_3}{k_2 + k_3}$$

The K values are the rate constants as defined in Figure 1. The lumped constant (LC) corrects for differences in the transport and phosphorylation rates of FDG and glucose, and is defined as the ratio of the FDG phosphorylation rate to the rate of glucose phosphorylation. It was set to 0.52 as previously measured for normal brain tissue (Reivich et al, 1985). $C_{pl}$ is the plasma glucose concentration (μmol ml$^{-1}$). Due to technical failure, the plasma glucose concentration measured at 50 min was not available for some studies ($n$ = 4 patients; 6 studies). No significant difference was seen between the available whole blood and plasma glucose concentrations measured at 20 min ($n$ = 11, mean difference ± standard deviation (s.d.): 0.07 ± 0.44; $t$-test $P > 0.2$, $t = 0.05$) (Bland and Altman, 1986). The 20 min whole blood glucose concentrations were used to compute MRGlu for this study.

### Calculation of standardized uptake values

In addition to calculating the glucose utilization rates for the regions defined, standardized uptake values (SUV) were also measured thereby allowing comparison of the two analysis methods. Standardized uptake values are a simplified model independent, semi-quantitative technique. It would be an easier calculation for clinical work being undertaken in busy nuclear medicine departments. It utilizes a static image, in these studies determined from the last 15 min of the scan, and normalizes it for known differences between the studies, i.e. patient weight or body surface area and injected activity of [$^{18}$F]FDG.

### Clinical response assessment

Contrast-enhanced CT scans and MRC neurological status assessment (Table 1) were performed pretreatment and after two courses

*© 2000 Cancer Research Campaign*

**Table 3** Glucose utilization rates (MRGlu μmol $min^{-1}$ $ml^{-1}$) for normal brain (B), whole tumour (WT) and high focal uptake area within tumour (HFU) defined on the pre- and post-treatment positron emission tomography scans

| | | MRGlu±SE | | | SUV | | | |
|---|---|---|---|---|---|---|---|---|
| **Pt** | **Tissue** | **Pre-Treatment** | **Post-Treatment** | **% Diff** | **Pre-Treatment** | **Post-Treatment** | **% Diff** | **Clinical Response (8 weeks)** |
| 1 | HFU | 0.39±0.02 | 0.35±0.04 | –10 | 182.930 | 183.222 | +0.2 | Control – |
| | WT | 0.27±0.02 | 0.28±0.01 | +4 | 143.964 | 139.096 | –3.4 | no |
| | B | 0.25±0.02 | 0.23±0.02 | –8 | 128.563 | 129.277 | +0.5 | treatment |
| 2 | HFU | 0.13±0.04 | 0.25±0.02 | +92* | NaN | 126.356 | NaN | |
| | WT | 0.14±0.05 | 0.16±0.02 | +14 | NaN | 94.827 | NaN | SD |
| | B | 0.35±0.02 | 0.31±0.02 | –11 | NaN | 178.134 | NaN | |
| 3 | HFU | 0.19±0.04 | 0.28±0.03 | +47* | 119.245 | 224.849 | +88.6 | |
| | WT | 0.31±0.02 | 0.34±0.02 | +10 | 167.278 | 242.294 | +44.8 | PD |
| | B | 0.30±0.05 | 0.37±0.03 | +23 | 145.595 | 242.283 | +39.9 | |
| 4 | HFU | 0.40±0.09 | 0.40±0.04 | 0 | 134.614 | 146.537 | +8.8 | |
| | WT | 0.26±0.08 | 0.31±0.01 | +19 | 133.101 | 138.403 | +4.0 | SD |
| | B | 0.30±0.02 | 0.26±0.02 | –13 | 146.871 | 143.182 | –2.5 | |
| 5 | HFU | 0.68±0.1 | 0.15±0.2 | –78* | 212.025 | 165.314 | –22 | |
| | WT | 0.29±0.03 | 0.31±0.03 | +7 | 177.381 | 127.662 | –28 | OR |
| | B | 0.21±0.03 | 0.22±0.02 | +5 | 195.105 | 131.321 | –32.6 | |
| 6 | HFU | 0.49±0.04 | 0.32±0.02 | –35* | 216.597 | 170.197 | –21.4 | |
| | WT | 0.37±0.03 | 0.27±0.01 | –27* | 159.408 | 134.394 | –15.7 | OR |
| | B | 0.36±0.05 | 0.37±0.01 | +3 | 136.467 | 174.204 | –27.7 | |
| 7 | HFU | 0.47±0.02 | 0.35±0.04 | –26 | 187.794 | 185.784 | –1.1 | |
| | WT | 0.34±0.01 | 0.36±0.02 | +6 | 168.519 | 185.118 | +9.8 | OR |
| | B | 0.37±0.02 | 0.36±0.02 | –3 | 182.040 | 197.707 | +8.6 | |
| 8 | HFU | 0.34±0.03 | 0.23±0.05 | –32* | 167.787 | 116.671 | –30.5 | |
| | WT | 0.29±0.01 | 0.16±0.03 | –45* | 136.021 | 107.034 | –21 | OR |
| | B | 0.28±0.03 | 0.33±0.02 | +18 | 145.106 | 124.000 | –14.5 | |
| 9 | HFU | 0.34±0.03 | 0.35±0.03 | +3 | 141.321 | 129.923 | +8.1 | |
| | WT | 0.39±0.03 | 0.33±0.02 | –15 | 146.839 | 118.619 | –19.2 | PD |
| | B | 0.24±0.03 | 0.23±0.02 | –4 | 127.998 | 114.384 | –10.6 | |

*Pre- and post-treatment MRGlu differ by at least 1 standard error (s.e.m.).

The percentage change (% diff) in glucose utilization and patient (Pt) clinical response assessed clinically and radiologically as progressive disease (PD), objective response (OR) or stable disease (SD) at 8 weeks is shown.

of temozolomide (8 weeks). For assessment of tumour response in high-grade glioma, objective response (OR) was used. This requires an improvement in the MRC neurological status by one grade and a clear-cut reduction in mass effect radiologically with a minimum duration of 4 weeks and no development or deterioration in other neurological symptoms or signs (Bleehen et al, 1989). Objective response was defined at 8 weeks. For analysis patients with stable and progressive disease were classified as non-responders.

### RESULTS

Details of patients are summarized in Table 2. All nine patients had received radiotherapy as their primary treatment (median 15 months previously; range 3.3–60.7 months). One (no. 4) had received adjuvant chemotherapy (temozolomide) combined with radiotherapy 2 years previously. All patients had normal liver and renal function prior to therapy and none had known diabetes mellitus. One patient (no. 8) had the second PET scan delayed until after the second course of temozolomide due to illness. The mean circulating whole blood glucose concentration at 20 min was (5.6±0.56 mmol $l^{-1}$).

Clinical response was available in all eight treated patients. Four patients had an objective response, two stable disease and two progressive disease at 8 weeks. The control patient was not assessed for clinical objective response.

Details of clinical and metabolic response for normal brain (B), whole tumour (WT) and tumour high focal (HFU) [$^{18}$F]FDG uptake are given in Table 3. The ROIs defined for each tissue area

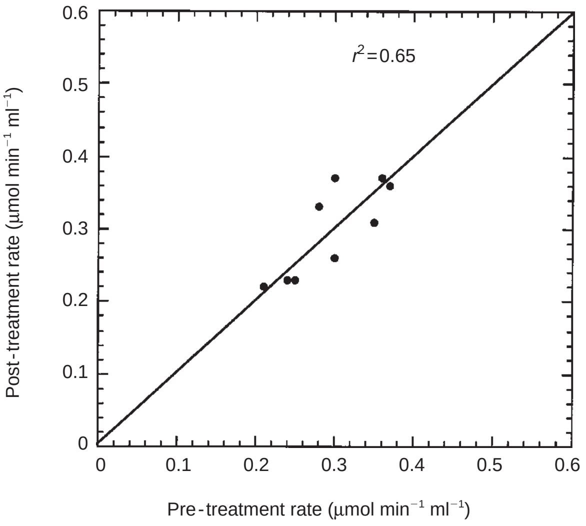

**Figure 2** Comparison of pre- and post-glucose utilization rates (MRGlu, μmol $min^{-1}$ $ml^{-1}$) for normal brain

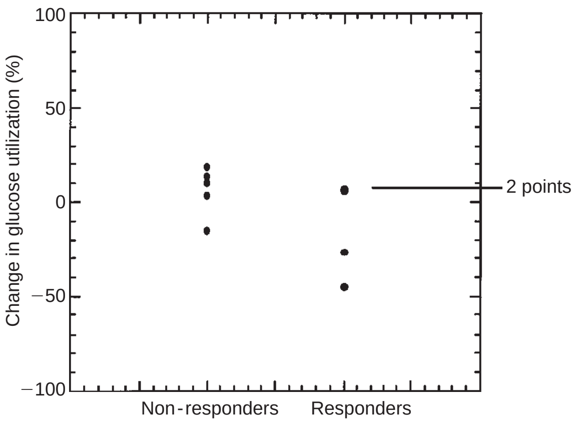

**Figure 3** Percentage changes in whole tumour (WT) glucose utilization rates (MRGlu μmol min$^{-1}$ ml$^{-1}$) compared with clinical response. There is no significant difference in percentage change in glucose utilization rates for the two groups ($n$ = 9, $t$-test $P < 0.1$)

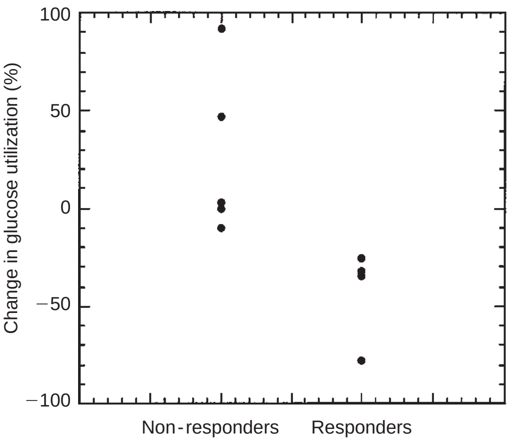

**Figure 4** Percentage changes in high focal tumour uptake (HFU) glucose utilization rates (MRGlu μmol min$^{-1}$ ml$^{-1}$) compared with clinical response. There is a significant difference between the responders and non-responders ($n$ = 9, $t$-test $P < 0.02$) which separates the groups

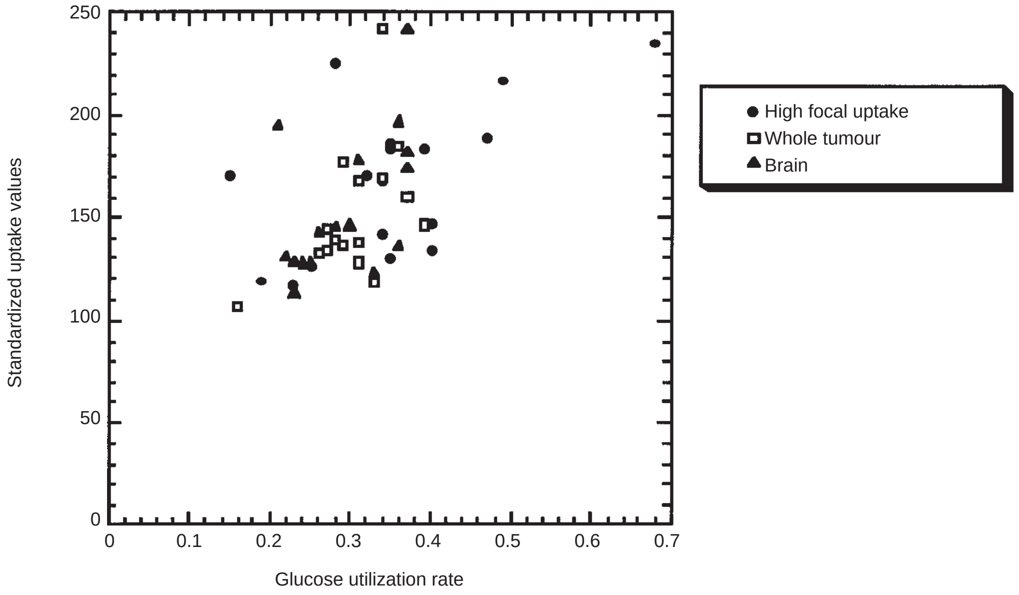

**Figure 5** Comparison of glucose utilization rates (MRGlu μmol min$^{-1}$ ml$^{-1}$) and standardized uptake values (SUV) for all tissue and tumour regions sampled contained a varying number of pixels since active tumours themselves are of varying sizes. Brain mean 223 pixels (range 113–432 pixels $n$ = 9); whole tumour, mean 1087 pixels (range 264–2673 pixels; $n$ = 9); high focal tumour uptake, mean 48 pixels (range 9–106 pixels; $n$ = 9).

For the control patient (no. 1) the percentage changes determined for sampled tissue regions were –8% for normal brain, +4% for whole tumour and –10% for the high uptake focus within the tumour.

Normal brain MRGlu was not significantly altered by treatment with temozolomide ($n$ = 9, $P > 0.2$ $t$-test) (Figure 2). Following temozolomide, the MRGlu for the whole tumour (WT) regions was decreased in three patients and increased in five patients. Alterations in whole tumour MRGlu did not consistently correspond with an objective response assessed at 8 weeks (Figure 3).

A greater than 25% reduction in [$^{18}$F]FDG uptake in the HFU regions was seen in four patients (nos 5–8). In the other four patients there was either no change or an increase in the MRGlu for the high focal uptake areas (nos 2, 3, 4, 9). Figure 4 shows the relationship between percentage change in MRGlu for HFU regions responding and non-responding patients. Those patients with 25% reduction in MRGlu at 14 days in HFU regions achieved an objective response at 8 weeks ($n$ = 4).

Pretreatment MRGlu levels in HFU were found to be related to response, being higher in the responding patient group. There was no difference between responders and non-responders in pre- or post-treatment normal brain MRGlu values or in absolute change in MRGlu.

Comparison of the MRGlu with SUV analysis for individual

 © 2000 Cancer Research Campaign

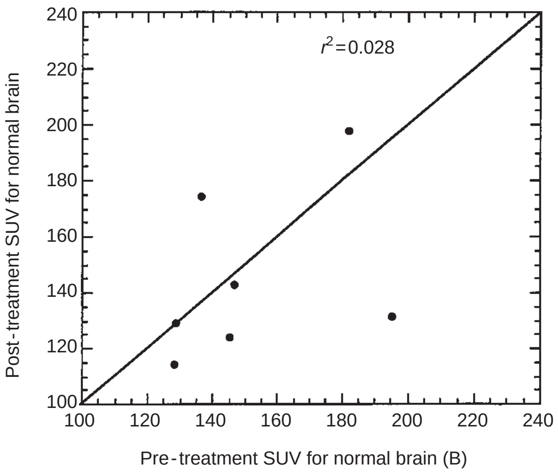

**Figure 6** Comparison of pre- and post-treatment standardized uptake values (SUV) for normal brain (B)

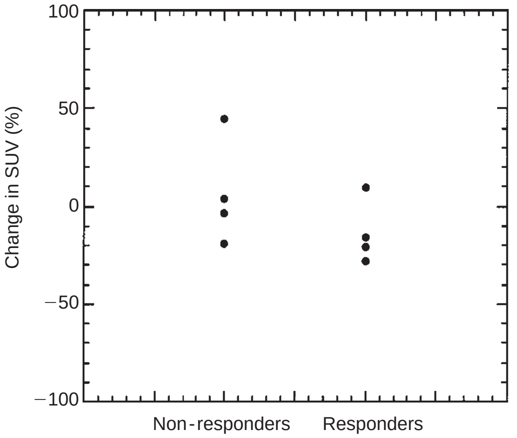

**Figure 7** Percentage changes in whole tumour (WT) standardized uptake values (SUV) compared with clinical response

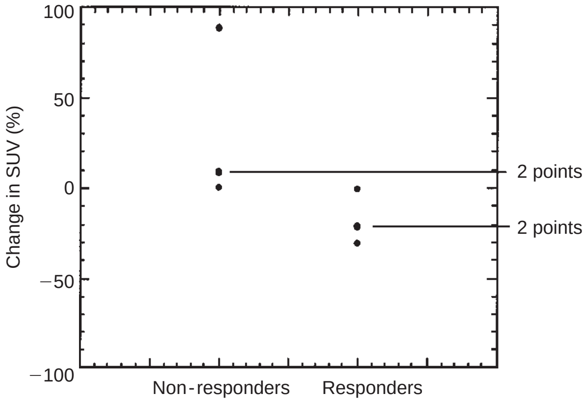

**Figure 8** Percentage changes in high focal tumour uptake (HFU) regions standardized uptake values (SUV) compared with clinical response patients for B, WT and HFU is shown in Figure 5. There were differences in normal brain SUV values following treatment with temozolomide (Figure 6). Comparison of percentage change in SUV following treatment with response category for both WT and HFU is shown in Figure 7 and 8 respectively. WT regions were not able to discriminate responders from non-responders. Percentage change in HFU regions was better at discriminating, but not as good as using MRGlu.

### DISCUSSION

In this study, reduction in MRGlu of > 25% in regions of HFU was seen 14 days after one cycle of temozolomide in patients who went on to have a objective response at 8 weeks.

Alterations in whole tumour MRGlu did not correspond with objective response. Despite visual comparison with anatomical imaging, whole tumour regions sampled may contain normal brain tissue, fibrosis, cystic fluid and oedema in addition to viable tumour cells. The MRGlu for whole tumour regions therefore may be, in part, determined by these non-tumour elements and will not be as affected by anti-proliferative chemotherapy as viable tumour. [$^{18}$F]FDG uptake is increased in viable tumour and regions of HFU probably contain a higher proportion of viable tumour cells.

Responding tumours had higher pretreatment MRGlu values in HFU regions. A high MRGlu is usually associated with more aggressive tumours (Di Chiro et al, 1982), suggesting that these may be more responsive to temozolomide treatment.

Blood–brain barrier disruption can alter the hexose transport system and allow passive diffusion of glucose and [$^{18}$F]FDG into brain tumours. It is unknown by how much or how quickly the integrity of the blood–brain barrier improves with response to therapy. However, independence of deoxyglucose utilization and blood–brain barrier disruption have been demonstrated in an animal glioma model (Blasberg et al, 1981). Alternations in tumour [$^{18}$F]FDG uptake were measured early in the course of treatment and probably reflect alterations in tumour [$^{18}$F]FDG utilization rather than changes in blood–brain barrier integrity.

As environmental conditions were not controlled for auditory and visual stimuli, the temporo-parietal brain was selected as a reference region for normal brain in this study. The variation in normal brain MRGlu was not significant within the precision of the measurement and therefore not attributable to treatment or environmental stimulation.

There is controversy over the optimal method to assess [$^{18}$F]FDG uptake. This study used MRGlu which has been the measurement standard for reporting [$^{18}$F]FDG uptake in brain tumours in the literature. The three-compartment model employed has been validated for use with [$^{18}$F]FDG in the normal human brain (Phelps et al, 1979) and the original model (Sokoloff et al, 1977) extended to include a dephosphorylation ($k_4$) rate constant (Phelps et al, 1979; Huang et al, 1980). It is thought that extrapolation of normal tissue data to tumours may not be entirely accurate since the model assumes tissue homogeneity (Herholz et al, 1990; Schmidt, 1992). The lumped constant for brain tumour has not yet been established (Fischman and Alpert, 1993). The lumped constant (LC 0.52) used in this study has been derived for normal brain (Reivich et al, 1985). Values derived for normal brain range from 0.42 to 0.86 (Phelps et al 1979; Reivich et al, 1985; Lammertsma et al, 1987) and where values have been reported for tumour they range from 0.72 to 3.10 (Spence et al, 1998). The absence of a consistent value for the lumped constant for tumour is a limitation for determination of MRGlu using [$^{18}$F]FDG. The LC

*© 2000 Cancer Research Campaign*

may vary following treatment, but is as likely to do so with normal brain as tumour. The use of percentage change in MRGlu circumvents the variation in the LC.

Standardized uptake values offer an alternative for the simplification of tumour [$^{18}$F]FDG uptake measurement and have previously been used in tumour response monitoring studies (Wahl et al, 1993). However, there are some disadvantages. SUV have been found to be positively correlated with body weight, due to reduced distribution of [$^{18}$F]FDG in the fat. Also, the underlying assumption that [$^{18}$F]FDG uptake is complete and irreversible at 60 min, the usual measurement time, is not usually fulfilled. Thus, measurement is often made in the initial uptake phase (Wahl et al, 1993; Hamberg et al, 1994). The differences demonstrated in normal brain SUV following treatment and the inability of changes in [$^{18}$F]FDG SUV to descriminate responders from non-responders as well as MRGlu suggests that the SUV measurement may be a less robust assessment for changes in tumour post-treatment. Brain tumour response is usually a small change and so the most sensitive measure is required to detect important small changes.

An assessment of the benefit of this method over simplified quantitation methods, e.g. SUV analysis, in terms of sensitivity and specificity warrants further assessment in studies specifically designed to address these issues.

The numbers for this pilot study are small and one control patient provides an indication, but not a measure of analysis technique reproducibility. Subsequent reproducibility [$^{18}$F]FDG PET studies are underway, of which currently three pairs have been performed. Similarly, a variation of $< 6.5\%$ for normal brain and $< 10.3\%$ for whole tumour has been measured (Brock et al, unpublished data).

In this pilot study [$^{18}$F]FDG-PET functional imaging provided a sensitive and quantitative measure of early tumour response to chemotherapy. The efficacy of this technique for quantifying degree of response is currently under evaluation in a larger group of patients with high-grade glioma receiving treatment with temozolomide, radiotherapy or a combination of the two. This will also define a more objective threshold value for the prediction of tumour response.

### ACKNOWLEDGEMENTS

Temozolomide was developed by the Cancer Research Campaign Phase I/II Sub Committee and is now licensed to Schering-Plough. This work was supported by the Cancer Research Campaign grant numbers SP193/0101 & 2 and a grant from Schering-Plough U.K. We are grateful to Mr A Blyth and Mr G Lewington for their technical assistance in performing these studies; to Jeff Yap, Vin Cunningham and Eric Aboagye for their helpful input and advice; and also to Jean Green for typing this manuscript.

### REFERENCES

Alavi JB, Alavi A, Chawluk J, Kushner M, Powe J, Hickey W and Reivich M (1988) Positron emission tomography in patients with glioma. A predictor of prognosis *Cancer* **62**: 1074–1078

Bennett MJ, Timperley WR, Taylor CB and Hill AS (1978) Isoenzymes of hexokinase in the developing, normal and neoplastic human brain. *Eur J Cancer* **14**: 189–193

Bland JM and Altman DG (1986) Statistical methods for assessing agreement between two methods of clinical measurement. *Lancet* **1**: 307–310

Blasberg RG, Groothuis DR and Molnar P (1981) The application of qualitative autoradiographic measurements in experimental brain tumours. *Semin Neurol* **1**: 203–333

Bleehen NM, Freedman LS and Stenning SP (1989) A randomized study of CCNU with and without benznidazole in the treatment of recurrent grades 3 and 4 astrocytoma. Report to the Medical Research Council by the Brain Tumor Working Party. *Int J Radiat Oncol Biol Phys* **16**: 1077–1081

Brown RS and Wahl RL (1993) Overexpression of Glut-1 glucose transporter in human breast cancer. An immunohistochemical study. *Cancer* **72**: 2979–2985

Brown RS, Leung JY, Fisher SJ, Frey KA, Ethier SP and Wahl RL (1996) Intratumoral distribution of tritiated-FDG in breast carcinoma: correlation between Glut-1 expression and FDG uptake. *J Nucl Med* **37**: 1042–1047

Cairncross JG, Pexman JH, Rathbone MP and DelMaestro RF (1985) Postoperative contrast enhancement in patients with brain tumor. *Ann Neurol* **17**: 570–572

Cairncross JG, Macdonald DR, Pexman JH and Ives FJ (1988) Steroid-induced CT changes in patients with recurrent malignant glioma. *Neurology* **38**: 724–726

De Witte O, Hildebrand J, Luxen A and Goldman S (1994) Acute effect of carmustine on glucose metabolism in brain and glioblastoma. *Cancer* **74**: 2836–2842

Di Chiro G, DeLaPaz RL, Brooks RA, Sokoloff L, Kornblith PL, Smith BH, Patronas NJ, Kuff GCV, Kessler RM, Johnson GS, Manning RG and Wolf AP (1982) Glucose utilization of cerebral gliomas measured by [$^{18}$F]-fluorodeoxyglucose and positron emission tomography. *Neurology* **32**: 1323–1329

Fischman AJ and Alpert NM (1993) FDG-PET in oncology: there's more to it than looking at pictures. *J Nucl Med* **34**: 6–11

Fulham MJ, Brunetti A, Aloj L, Raman R, Dwyer AJ and Di Chiro G (1995) Decreased cerebral glucose metabolism in patients with brain tumours: an effect of cortocosteroids. *J Neurosurg* **83**: 657–664

Hamacher K, Coenen HH and Stocklin G (1986) Efficient stereospecific synthesis of no-carrier-added 2-[$^{18}$F]-fluoro-2-deoxy-D-glucose using aminopolyether supported nucleophilic substitution. *J Nucl Med* **27**: 235–238

Hamberg LM, Hunter GJ, Alpert NM, Choi NC, Babich JW and Fischman AJ (1994) The dose uptake ratio as an index of glucose metabolism: useful parameter or oversimplification? *J Nucl Med* **35**: 1308–1312

Herholz K, Wienhard K and Heiss WD (1990) Validity of PET studies in brain tumors. *Cerebrovasc Brain Metab Rev* **2**: 240–265

Herholz K, Rudolf J and Heiss WD (1992) FDG transport and phosphorylation in human gliomas measured with dynamic PET. *J Neurosurg* **12**: 159–165.

Herholz K, Pietrzyk U, Voges J, Schroder R, Halber M, Treuer H, Sturm V and Heiss WD (1993) Correlation of glucose consumption and tumour cell density in astrocytomas. A stereotactic PET study. *J Neurosurg* **79**: 853–858

Higashi K, Clavo AC and Wahl RL (1993) Does FDG uptake measure proliferative activity of human cancer cells? In vitro comparison with DNA flow cytometry and tritiated thymidine uptake [see comments]. *J Nucl Med* **34**: 414–419

Holthoff VA, Herholz K, Berthold F, Widemann B, Schroder R, Neubauer I and Heiss WD (1993) In vivo metabolism of childhood posterior fossa tumors and primitive neuroectodermal tumors before and after treatment. *Cancer* **72**: 1394–1403

Huang SC, Phelps ME, Hoffman EJ, Sideris K, Selin CJ and Kuhl DE (1980) Noninvasive determination of local cerebral metabolic rate of glucose in man. *Am J Physiol* **238**: E69–82

Ichiya Y, Kuwabara Y, Otsuka M, et al. (1991) Assessment of response to cancer therapy using fluorine-18-fluorodeoxyglucose and positron emission tomography. *J Nucl Med* **32**: 1655–1660

Ishizu K, Nishizawa S, Yonekura Y, Sadato N, Magata Y, Tamaki N, Tsuchida T, Okazawg H, Miyatake S, Ishikawa M et al. (1994) Effects of hyperglycemia on FDG uptake in human brain and glioma [see comments]. *J Nucl Med* **35**: 1104–1109

Jansson T, Westlin JE, Ahlstrom H, Lila A, Langstrom B and Bergh J (1995) Positron emission tomography studies in patients with locally advanced and/or metastatic breast cancer: a method for early therapy evaluation? *J Clin Oncol* **13**: 1470–1477

Lammertsma AA, Brooks DJ, Frackowiak RS, Beaney RP, Herold S, Heather JD, Palmer AJ and Jones T (1987) Measurement of glucose utilisation with [$^{18}$F]2-fluoro-2-deoxy-D-glucose: a comparison of different analytical methods. *J Cereb Blood Flow Metab* **7**: 161–172

Lindholm P, Minn H, Leskinen KS, Bergman J, Ruotsalainen U and Joensuu H (1993) Influence of the blood glucose concentration on FDG uptake in cancer – a PET study [see comments]. *J Nucl Med* **34**: 1–6

Minn H, Joensuu H, Ahonen A and Klemi P (1988a) Fluorodeoxyglucose imaging: a method to assess the proliferative activity of human cancer in vivo. Comparison with DNA flow cytometry in head and neck tumours. *Cancer* **61**: 1776–1781

 © 2000 Cancer Research Campaign

Minn H, Paul R and Ahonen A (1988b) Evaluation of treatment response to radiotherapy in head and neck cancer with fluorine-18 fluorodeoxyglucose. *J Nucl Med* **29**: 1521–1525

Newlands ES, Blackledge GR, Slack JA, Rustin GJ, Smith DB, Stuart NS, Quarterman CP, Hoffman R, Stevens MF, Brampton MH and Gibson AC (1992) Phase I trial of temozolomide (CCRG 81045: M&B 39831: NSC 362856). *Br J Cancer* **65**: 287–291

Okada J, Yoshikawa K, Itami M, Imaseki K, Uno K, Itami J, Kuyama J, Mikata A and Arimizu N (1992) Positron emission tomography using fluorine-18-fluorodeoxyglucose in malignant lymphoma: a comparison with proliferative activity. *J Nucl Med* **33**: 325–329

O'Reilly SM, Newlands ES, Glaser MG, Brampton M, Rice Edwards JM, Illingworth RD, Richards P, Kennard C, Colquhoun T and Lewis P (1993) Temozolomide: a new oral cytotoxic chemotherapeutic agent with promising activity against primary brain tumours [published erratum appears in *Eur J Cancer* **29a**: 1500]. *Eur J Cancer* **29a**: 940–942

Patronas NJ, Di Chiro G, Brooks RA, DeLaPaz RL, Kornblith PL, Smith BH, Rizzoli HV, Kessler RM, Manning RG, Channing M, Wolf AP and O'Connor CM (1982) Work in progress: [$^{18}$F]fluorodeoxyglucose and positron emission tomography in the evaluation of radiation necrosis of the brain. *Radiology* **144**: 885–889

Phelps ME, Huang SC, Hoffman EJ, Selin C, Sokoloff L and Kuhl DE (1979) Tomographic measurement of local cerebral glucose metabolic rate in humans with (F-18)2-fluoro-2-deoxy-D-glucose: validation of method. *Ann Neurol* **6**: 371–388

Price P and Jones T (1995) Can positron emission tomography (PET) be used to detect subclinical response to cancer therapy? The EC PET Oncology Concerted Action and the EORTC PET Study Group. *Eur J Cancer* **31a**: 1924–1927

Ranicar AS, Williams CW, Schnorr L, Clark JC, Rhodes CG, Bloomfield PM and Jones T (1991) The on-line monitoring of continuously withdrawn arterial blood during PET studies using a single BGO/photomultiplier assembly and non-stick tubing. *Med Prog Technol* **17**: 259–264

Reivich M, Alavi A, Wolf A, Fowler J, Russell J, Arnett C, MacGregor RR, Shive CY, Atkins H, Anand A, Dann R and Greenberg JH (1985) Glucose metabolic rate kinetic model parameter determination in humans: the lumped constants and rate constants for [$^{18}$F]fluorodeoxyglucose and [$^{11}$C]deoxyglucose. *J Cereb Blood Flow Metab* **5**: 179–192

Roelcke U, Blasberg RG, von Ammor K, Hofer S, Vontobel P, Maguire RP, Radii EW, Herman RP and Leenders KL (1998) Dexamethasone treatment and plasma glucose values: relevance for fluorine-18-fluorodeoxyglucose uptake measurements in gliomas. *J Nucl Med* **39**: 879–884

Rozental JM, Levin RL, Nickles RJ and Dobkin JA (1989) Glucose uptake by gliomas after treatment. A positron emission tomographic study [see comments]. *Arch Neurol* **46**: 1302–1307

Schmidt K, Lucignani G, Moresco RM, Rizzo G, Gilardi MC, Messa C, Colombo F, Fazio F and Sokoloff L (1992) Errors introduced by tissue heterogeneity in estimation of local cerebral glucose utilization with current kinetic models of the [$^{18}$F]fluorodeoxyglucose method. *J Cereb Blood Flow Metab* **12**: 823–834

Sokoloff L, Reivich M, Kennedy C, Des Rosiers MH, Patlak CS, Pettigrew KD, Sakurada O and Shinohara M (1977) The [$^{14}$C]deoxyglucose method for the measurement of local cerebral glucose utilization: theory, procedure, and normal values in the conscious and anesthetized albino rat. *J Neurochem* **28**: 897–916

Spence AM, Muzi M, Graham MM et al. (1998) Glucose metabolism in human malignant gliomas measured quantitatively with PET, 1-[C-11]Glucose and FDG: analysis of the FDG lumped constant. *J Nucl Med* **39**: 440–448

Valk PE, Budinger TF, Levin VA, Silver P, Gutin PH and Doyle WL (1988) PET of malignant cerebral tumors after interstitial brachytherapy. Demonstration of metabolic activity and correlation with clinical outcome. *J Neurosurg* **69**: 830–838

Wahl RL, Zasadny K, Helvie M, Hutchins GD, Weber B and Cody R (1993) Metabolic monitoring of breast cancer chemohormonotherapy using positron emission tomography: initial evaluation. *J Clin Oncol* **11**: 2101–2111

Woods RP, Cherry SR and Mazziotta JC (1992) Rapid automated alogorithm for aligning and reslicing PET images. *J Comput A Tomogr* **16**: 620–633

*© 2000 Cancer Research Campaign*
